# *N*-(2,6-Dimethyl­phen­yl)-2-(2-thien­yl)acetamide

**DOI:** 10.1107/S1600536809049782

**Published:** 2009-11-25

**Authors:** Marcelle Ferreira de Lima, Marcus V. N. de Souza, Edward R. T. Tiekink, James L. Wardell, Solange M. S. V. Wardell

**Affiliations:** a FioCruz – Fundação Oswaldo Cruz, Instituto de Tecnologia em Farmacos–FarManguinhos, Rua Sizenando Nabuco, 100, Manguinhos, 21041-250 Rio de Janeiro, RJ, Brazil; b Department of Chemistry, University of Malaya, 50603 Kuala Lumpur, Malaysia; c Centro de Desenvolvimento Tecnológico em Saúde (CDTS), Fundação Oswaldo Cruz (FIOCRUZ), Casa Amarela, Campus de Manguinhos, Av. Brasil 4365, 21040-900 Rio de Janeiro, RJ, Brazil; d CHEMSOL, 1 Harcourt Road, Aberdeen AB15 5NY, Scotland

## Abstract

The thienyl ring in the title compound, C_14_H_15_NOS, is disordered over two diagonally opposite positions, the major component having a site-occupancy factor of 0.569 (3). The mol­ecule is highly twisted with respect to the central amide group, which is reflected in the dihedral angle formed between the thienyl and benzene rings of 77.01 (15)° [70.34 (18)° for the minor component]. In the crystal, mol­ecules self-associate into chains along [100] *via* N—H⋯O hydrogen bonds. The chains are reinforced by complementary C—H⋯O contacts.

## Related literature

For a general overview of 2-substituted thio­phenes, see: Campaigne (1984 ▶); Kleemann *et al.* (2006 ▶). For recent bio­logical studies on 2-substituted thio­phenes, see: Lourenço *et al.* (2007 ▶).
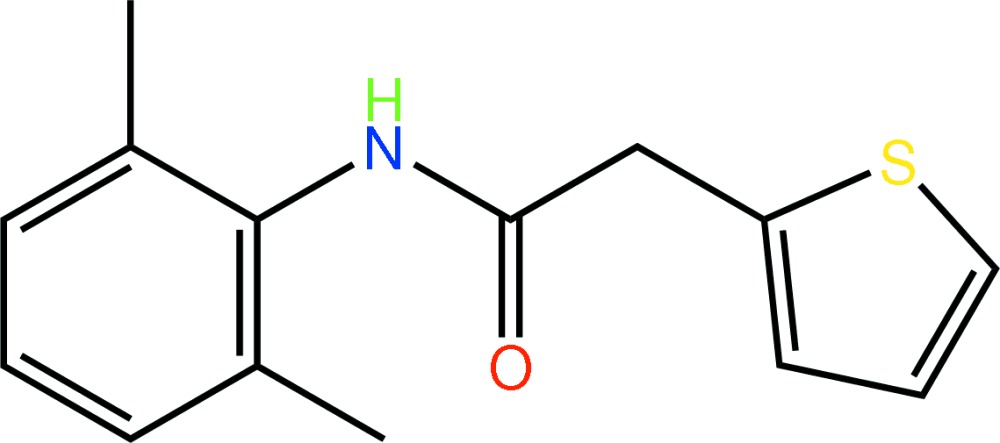


## Experimental

### 

#### Crystal data

C_14_H_15_NOS
*M* *_r_* = 245.34
Triclinic, 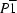

*a* = 4.7489 (2) Å
*b* = 11.7309 (5) Å
*c* = 11.8117 (4) Å
α = 100.981 (2)°
β = 95.427 (2)°
γ = 101.104 (2)°
*V* = 627.96 (4) Å^3^
*Z* = 2
Mo *K*α radiation
μ = 0.24 mm^−1^
*T* = 120 K
0.16 × 0.06 × 0.02 mm

#### Data collection

Nonius KappaCCD area-detector diffractometer
Absorption correction: multi-scan (*SADABS*; Sheldrick, 2003 ▶) *T*_min_ = 0.896, *T*_max_ = 1.000
11243 measured reflections
2840 independent reflections
2388 reflections with *I* > 2σ(*I*)
*R*_int_ = 0.043

#### Refinement

*R*[*F* ^2^ > 2σ(*F* ^2^)] = 0.047
*wR*(*F* ^2^) = 0.121
*S* = 1.04
2840 reflections
175 parameters
H-atom parameters constrained
Δρ_max_ = 0.27 e Å^−3^
Δρ_min_ = −0.21 e Å^−3^

### 

Data collection: *COLLECT* (Hooft, 1998 ▶); cell refinement: *DENZO* (Otwinowski & Minor, 1997 ▶) and *COLLECT*; data reduction: *DENZO* and *COLLECT*; program(s) used to solve structure: *SHELXS97* (Sheldrick, 2008 ▶); program(s) used to refine structure: *SHELXL97* (Sheldrick, 2008 ▶); molecular graphics: *DIAMOND* (Brandenburg, 2006 ▶); software used to prepare material for publication: *publCIF* (Westrip, 2009 ▶).

## Supplementary Material

Crystal structure: contains datablocks global, I. DOI: 10.1107/S1600536809049782/hg2603sup1.cif

Structure factors: contains datablocks I. DOI: 10.1107/S1600536809049782/hg2603Isup2.hkl

Additional supplementary materials:  crystallographic information; 3D view; checkCIF report

## Figures and Tables

**Table 1:** Hydrogen-bond geometry (Å, °)

*D*—H⋯*A*	*D*—H	H⋯*A*	*D*⋯*A*	*D*—H⋯*A*
N1—H1⋯O1^i^	0.88	2.04	2.8701 (18)	157
C5—H5b⋯O1^i^	0.99	2.38	3.2622 (19)	148

Symmetry code: (i) 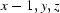.

**Fig. 1.**
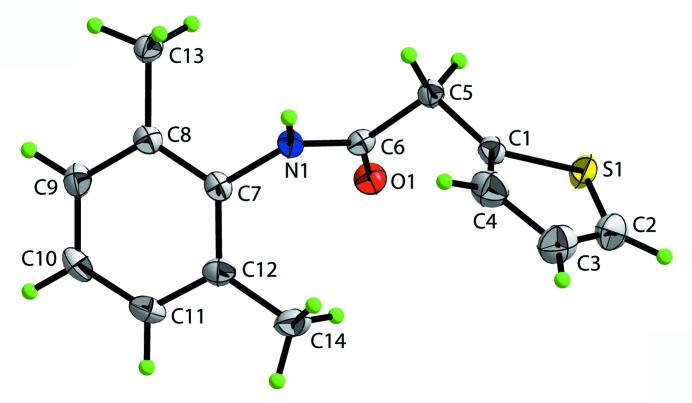
Molecular structure (I) showing atom-labelling scheme and displacement ellipsoids at the 50% probability level. Only the major component of the disordered thienyl ring is shown for reasons of clarity.

**Fig. 2.**
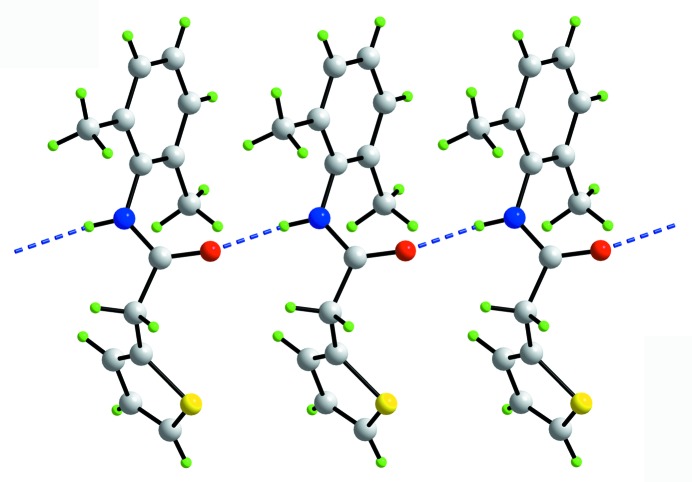
Supramolecular chain in (I) mediated by N–H···O hydrogen bonds (blue dashed lines). Colour code: S, yellow; O, red; N, blue; C, grey; and H, green.

## supplementary crystallographic information

### Comment

2-Substituted thiophenes have been found to have various uses, for example as dyestuffs, flavour agents, drugs, and inhibitors (Campaigne, 1984). Indeed, thiophenes are present in many natural and synthetic products with a wide range of pharmacological activities (Kleemann *et al.*, 2006). The in vitro anti-mycobacterial activities of a series of *N*-(aryl)-2-thiophen-2-ylacetamide derivatives were recently investigated (Lourenço *et al.*, 2007): encouraging activities were detected for some derivatives. The search for new drugs having anti-bacterial activity against Mycobacterium tuberculosis is a vital task due to the increase of multi-drug resistant tuberculosis (MDR-TB) and AIDS cases worldwide and the increasing resistance to the currently used main line drugs such as isoniazid and rifampin (http://www.who.int/tdr/diseases/tb/default.htm). It was in this context that the title compound, (I), was synthesized.

The central O1, N1, C5 and C6 moiety in (I), Fig. 1, is planar with the maximum deviation from the least-squares plane through these atoms being 0.0072 (13) Å for the C6 atom. Otherwise, the molecule is highly twisted as seen in the values of the S1–C1–C5–C6 and C6–N1–C7–C8 torsion angles of 102.98 (15) ° (-69.07 (18) ° for the minor component of the thienyl ring) and -116.27 (17) °, respectively. The dihedral angle between the planes through the thienyl and benzene rings are 77.01 (15) and 70.34 (18) ° for the major and minor components of the disordered thienyl ring, respectively.

In the crystal structure, molecules are connected into linear supramolecular chains *via* C(4), {···HNC(═O)}, synthons, Table 1 and Fig. 2. Chains, which are aligned along [1 0 0], are reinforced by complementary C5–H···O1 contacts, Table 1, so that the acceptor carbonyl-O1 atom is bifurcated.

### Experimental

A solution of 2,6-dimethylaniline (2 mmol) and 2-thienylacetyl chloride (2 mmol) in tetrahydrofuran (20 ml), was stirred for 2 h at room temperature, water (30 ml) added and the mixture was extracted with ethyl acetate (2 *x* 20 ml). The combined organic layers were washed with saturated aqueous NaHCO_3_ and brine, dried over MgSO_4_, filtered, and rotary evaporated to give the crude product, (yield 90%) which was recrystallized twice from EtOH. m. pt.: 405–406 K; CG/MS: m/*z* [*M*]+.: 245. ^1^H NMR [500.00 MHz, DMSO-d_6_] δ: 9.46 (s, 1H, NH), 7.38 (dd, 1H, J = 6.5, 2.0 Hz), 7.05–6.97 (m, 5H), 3.82 (s, 2H, CH_2_CO), 2.09 (s, 6H, Me) p.p.m. ^13^C NMR (125.0 MHz, DMSO-d_6_) δ: 167.7, 137.6, 135.1, 134.8, 127.6, 126.6, 126.4, 126.2, 124.8, 36.5, 17.9 p.p.m. IR (KBr, cm^-1^): ν_max_ 1644 (CO).

### Refinement

All H atoms were geometrically placed (N–H = 0.88 Å and C–H = 0.95–0.99 Å) and refined as riding with *U*_iso_(H) = 1.2–1.5*U*_eq_(N, C). The thienyl ring was disordered with two diagonally opposed positions resolved for the S1 and C4 atoms. The major component had a site occupancy factor = 0.569 (3).

### Crystal data

**Table:**

C_14_H_15_NOS	*Z* = 2
*M**_r_* = 245.34	*F*(000) = 260
Triclinic, *P*1	*D*_x_ = 1.298 Mg m^−^^3^
Hall symbol: -P 1	Mo *K*α radiation, λ = 0.71073 Å
*a* = 4.7489 (2) Å	Cell parameters from 2752 reflections
*b* = 11.7309 (5) Å	θ = 2.9–27.5°
*c* = 11.8117 (4) Å	µ = 0.24 mm^−^^1^
α = 100.981 (2)°	*T* = 120 K
β = 95.427 (2)°	Block, pale-brown
γ = 101.104 (2)°	0.16 × 0.06 × 0.02 mm
*V* = 627.96 (4) Å^3^	

### Data collection

**Table:**

Nonius KappaCCD area-detector diffractometer	2840 independent reflections
Radiation source: Enraf Nonius FR591 rotating anode	2388 reflections with *I* > 2σ(*I*)
10 cm confocal mirrors	*R*_int_ = 0.043
Detector resolution: 9.091 pixels mm^-1^	θ_max_ = 27.5°, θ_min_ = 3.6°
φ and ω scans	*h* = −6→6
Absorption correction: multi-scan (*SADABS*; Sheldrick, 2003)	*k* = −15→15
*T*_min_ = 0.896, *T*_max_ = 1.000	*l* = −14→15
11243 measured reflections	

### Refinement

**Table:**

Refinement on *F*^2^	Primary atom site location: structure-invariant direct methods
Least-squares matrix: full	Secondary atom site location: difference Fourier map
*R*[*F*^2^ > 2σ(*F*^2^)] = 0.047	Hydrogen site location: inferred from neighbouring sites
*wR*(*F*^2^) = 0.121	H-atom parameters constrained
*S* = 1.04	*w* = 1/[σ^2^(*F*_o_^2^) + (0.0468*P*)^2^ + 0.4276*P*] where *P* = (*F*_o_^2^ + 2*F*_c_^2^)/3
2840 reflections	(Δ/σ)_max_ < 0.001
175 parameters	Δρ_max_ = 0.27 e Å^−^^3^
0 restraints	Δρ_min_ = −0.21 e Å^−^^3^

### Special details

**Table:**

Geometry. All s.u.'s (except the s.u. in the dihedral angle between two l.s. planes) are estimated using the full covariance matrix. The cell s.u.'s are taken into account individually in the estimation of s.u.'s in distances, angles and torsion angles; correlations between s.u.'s in cell parameters are only used when they are defined by crystal symmetry. An approximate (isotropic) treatment of cell s.u.'s is used for estimating s.u.'s involving l.s. planes.
Refinement. Refinement of *F*^2^ against ALL reflections. The weighted *R*-factor *wR* and goodness of fit *S* are based on *F*^2^, conventional *R*-factors *R* are based on *F*, with *F* set to zero for negative *F*^2^. The threshold expression of *F*^2^ > 2σ(*F*^2^) is used only for calculating *R*-factors(gt) *etc*. and is not relevant to the choice of reflections for refinement. *R*-factors based on *F*^2^ are statistically about twice as large as those based on *F*, and *R*- factors based on ALL data will be even larger.

### Fractional atomic coordinates and isotropic or equivalent isotropic displacement parameters (Å^2^)

**Table:**

	*x*	*y*	*z*	*U*_iso_*/*U*_eq_	Occ. (<1)
O1	0.5541 (2)	0.54877 (11)	0.35576 (11)	0.0269 (3)	
N1	0.1431 (3)	0.61274 (11)	0.31337 (12)	0.0198 (3)	
H1	−0.0473	0.5949	0.3056	0.024*	
C1	0.0819 (3)	0.31943 (14)	0.26257 (15)	0.0227 (3)	0.569 (3)
S1	0.2856 (3)	0.21398 (11)	0.27132 (12)	0.0304 (3)	0.569 (3)
C2	0.1372 (5)	0.14166 (18)	0.13388 (18)	0.0401 (5)	0.569 (3)
H2	0.1810	0.0680	0.1000	0.048*	0.569 (3)
C3	−0.0428 (5)	0.19078 (19)	0.0743 (2)	0.0431 (5)	0.569 (3)
H3	−0.1283	0.1653	−0.0048	0.052*	0.569 (3)
C4	−0.0767 (15)	0.2938 (6)	0.1609 (6)	0.0469 (15)	0.569 (3)
H4	−0.2108	0.3405	0.1432	0.056*	0.569 (3)
C1'	0.0819 (3)	0.31943 (14)	0.26257 (15)	0.0227 (3)	0.431 (3)
S1'	−0.1147 (5)	0.32153 (18)	0.13045 (17)	0.0394 (5)	0.431 (3)
C2'	−0.0428 (5)	0.19078 (19)	0.0743 (2)	0.0431 (5)	0.431 (3)
H2'	−0.1318	0.1500	−0.0018	0.052*	0.431 (3)
C3'	0.1372 (5)	0.14166 (18)	0.13388 (18)	0.0401 (5)	0.431 (3)
H3'	0.2157	0.0741	0.1085	0.048*	0.431 (3)
C4'	0.1802 (19)	0.2208 (7)	0.2491 (7)	0.0390 (18)	0.431 (3)
H4'	0.2787	0.2009	0.3140	0.047*	0.431 (3)
C5	0.1040 (3)	0.42001 (14)	0.36525 (14)	0.0216 (3)	
H5A	0.1911	0.3995	0.4362	0.026*	
H5B	−0.0922	0.4326	0.3774	0.026*	
C6	0.2889 (3)	0.53354 (14)	0.34521 (13)	0.0199 (3)	
C7	0.2832 (3)	0.72479 (14)	0.29151 (14)	0.0203 (3)	
C8	0.2477 (3)	0.82854 (14)	0.36467 (14)	0.0220 (3)	
C9	0.3851 (4)	0.93774 (15)	0.34462 (16)	0.0279 (4)	
H9	0.3609	1.0093	0.3922	0.033*	
C10	0.5560 (4)	0.94333 (16)	0.25656 (17)	0.0313 (4)	
H10	0.6530	1.0183	0.2453	0.038*	
C11	0.5854 (4)	0.83976 (17)	0.18487 (16)	0.0308 (4)	
H11	0.7023	0.8446	0.1243	0.037*	
C12	0.4471 (4)	0.72805 (16)	0.19947 (14)	0.0254 (4)	
C13	0.0684 (4)	0.82262 (15)	0.46287 (15)	0.0261 (4)	
H13A	−0.1323	0.7830	0.4311	0.039*	
H13B	0.0740	0.9034	0.5059	0.039*	
H13C	0.1469	0.7776	0.5155	0.039*	
C14	0.4706 (4)	0.61673 (17)	0.11603 (16)	0.0339 (4)	
H14A	0.5533	0.6381	0.0481	0.051*	
H14B	0.2776	0.5652	0.0909	0.051*	
H14C	0.5962	0.5745	0.1549	0.051*	

### Atomic displacement parameters (Å^2^)

**Table:**

	*U*^11^	*U*^22^	*U*^33^	*U*^12^	*U*^13^	*U*^23^
O1	0.0159 (5)	0.0303 (6)	0.0356 (7)	0.0055 (5)	0.0035 (5)	0.0094 (5)
N1	0.0142 (6)	0.0179 (6)	0.0263 (7)	0.0014 (5)	0.0030 (5)	0.0043 (5)
C1	0.0207 (7)	0.0205 (8)	0.0270 (8)	0.0030 (6)	0.0047 (6)	0.0062 (6)
S1	0.0376 (7)	0.0255 (5)	0.0302 (6)	0.0155 (5)	0.0043 (5)	0.0023 (4)
C2	0.0515 (12)	0.0277 (10)	0.0373 (11)	0.0033 (9)	0.0092 (9)	0.0013 (8)
C3	0.0420 (11)	0.0397 (11)	0.0403 (11)	0.0010 (9)	0.0047 (9)	−0.0009 (9)
C4	0.056 (3)	0.041 (3)	0.044 (4)	0.019 (2)	−0.007 (2)	0.009 (2)
C1'	0.0207 (7)	0.0205 (8)	0.0270 (8)	0.0030 (6)	0.0047 (6)	0.0062 (6)
S1'	0.0444 (8)	0.0388 (10)	0.0304 (10)	0.0132 (7)	−0.0092 (7)	−0.0009 (6)
C2'	0.0420 (11)	0.0397 (11)	0.0403 (11)	0.0010 (9)	0.0047 (9)	−0.0009 (9)
C3'	0.0515 (12)	0.0277 (10)	0.0373 (11)	0.0033 (9)	0.0092 (9)	0.0013 (8)
C4'	0.040 (4)	0.044 (4)	0.036 (4)	0.011 (3)	0.002 (3)	0.014 (3)
C5	0.0204 (7)	0.0190 (7)	0.0258 (8)	0.0043 (6)	0.0043 (6)	0.0051 (6)
C6	0.0184 (7)	0.0213 (7)	0.0188 (7)	0.0042 (6)	0.0030 (6)	0.0015 (6)
C7	0.0165 (7)	0.0204 (8)	0.0229 (8)	0.0016 (6)	−0.0004 (6)	0.0059 (6)
C8	0.0182 (7)	0.0226 (8)	0.0248 (8)	0.0033 (6)	0.0017 (6)	0.0059 (6)
C9	0.0275 (8)	0.0218 (8)	0.0345 (9)	0.0047 (7)	0.0040 (7)	0.0073 (7)
C10	0.0306 (9)	0.0266 (9)	0.0372 (10)	−0.0013 (7)	0.0058 (8)	0.0146 (8)
C11	0.0309 (9)	0.0358 (10)	0.0270 (9)	0.0022 (7)	0.0095 (7)	0.0125 (8)
C12	0.0238 (8)	0.0298 (9)	0.0224 (8)	0.0053 (7)	0.0028 (6)	0.0060 (7)
C13	0.0241 (8)	0.0217 (8)	0.0321 (9)	0.0038 (6)	0.0088 (7)	0.0033 (7)
C14	0.0389 (10)	0.0379 (10)	0.0237 (9)	0.0065 (8)	0.0103 (8)	0.0024 (8)

### Geometric parameters (Å, °)

**Table:**

O1—C6	1.2285 (19)	C4'—H4'	0.9500
N1—C6	1.347 (2)	C5—C6	1.520 (2)
N1—C7	1.4360 (19)	C5—H5A	0.9900
N1—H1	0.8800	C5—H5B	0.9900
C1—C4	1.305 (7)	C7—C12	1.398 (2)
C1—C5	1.502 (2)	C7—C8	1.401 (2)
C1—S1	1.723 (2)	C8—C9	1.395 (2)
S1—C2	1.697 (2)	C8—C13	1.507 (2)
C2—C3	1.337 (3)	C9—C10	1.382 (3)
C2—H2	0.9500	C9—H9	0.9500
C3—C4	1.472 (7)	C10—C11	1.382 (3)
C3—H3	0.9500	C10—H10	0.9500
C4—H4	0.9500	C11—C12	1.398 (2)
C1'—C4'	1.317 (9)	C11—H11	0.9500
C1'—C5	1.502 (2)	C12—C14	1.509 (2)
C1'—S1'	1.748 (3)	C13—H13A	0.9800
S1'—C2'	1.663 (3)	C13—H13B	0.9800
C2'—C3'	1.337 (3)	C13—H13C	0.9800
C2'—H2'	0.9500	C14—H14A	0.9800
C3'—C4'	1.466 (8)	C14—H14B	0.9800
C3'—H3'	0.9500	C14—H14C	0.9800
			
C6—N1—C7	123.24 (13)	C6—C5—H5B	109.6
C6—N1—H1	118.4	H5A—C5—H5B	108.1
C7—N1—H1	118.4	O1—C6—N1	123.38 (15)
C4—C1—C5	130.6 (3)	O1—C6—C5	120.78 (14)
C4—C1—S1	109.9 (3)	N1—C6—C5	115.82 (13)
C5—C1—S1	119.57 (13)	C12—C7—C8	122.10 (14)
C2—S1—C1	89.60 (12)	C12—C7—N1	120.16 (14)
C3—C2—S1	118.43 (17)	C8—C7—N1	117.74 (13)
C3—C2—H2	120.8	C9—C8—C7	118.09 (15)
S1—C2—H2	120.8	C9—C8—C13	120.84 (15)
C2—C3—C4	103.3 (3)	C7—C8—C13	121.07 (14)
C2—C3—H3	128.3	C10—C9—C8	120.92 (16)
C4—C3—H3	128.3	C10—C9—H9	119.5
C1—C4—C3	118.4 (5)	C8—C9—H9	119.5
C1—C4—H4	120.8	C11—C10—C9	119.88 (16)
C3—C4—H4	120.8	C11—C10—H10	120.1
C4'—C1'—C5	132.7 (4)	C9—C10—H10	120.1
C4'—C1'—S1'	108.1 (4)	C10—C11—C12	121.52 (16)
C5—C1'—S1'	119.22 (13)	C10—C11—H11	119.2
C2'—S1'—C1'	88.81 (14)	C12—C11—H11	119.2
C3'—C2'—S1'	121.55 (19)	C11—C12—C7	117.44 (16)
C3'—C2'—H2'	119.2	C11—C12—C14	120.33 (15)
S1'—C2'—H2'	119.2	C7—C12—C14	122.21 (15)
C2'—C3'—C4'	100.8 (4)	C8—C13—H13A	109.5
C2'—C3'—H3'	129.6	C8—C13—H13B	109.5
C4'—C3'—H3'	129.6	H13A—C13—H13B	109.5
C1'—C4'—C3'	119.9 (6)	C8—C13—H13C	109.5
C1'—C4'—H4'	120.1	H13A—C13—H13C	109.5
C3'—C4'—H4'	120.1	H13B—C13—H13C	109.5
C1'—C5—C6	110.43 (13)	C12—C14—H14A	109.5
C1—C5—C6	110.43 (13)	C12—C14—H14B	109.5
C1'—C5—H5A	109.6	H14A—C14—H14B	109.5
C1—C5—H5A	109.6	C12—C14—H14C	109.5
C6—C5—H5A	109.6	H14A—C14—H14C	109.5
C1'—C5—H5B	109.6	H14B—C14—H14C	109.5
C1—C5—H5B	109.6		
			
C4—C1—S1—C2	−0.1 (4)	C7—N1—C6—C5	179.66 (13)
C5—C1—S1—C2	179.05 (14)	C1—C5—C6—O1	−76.75 (19)
C1—S1—C2—C3	4.17 (19)	C1—C5—C6—N1	101.88 (16)
S1—C2—C3—C4	−6.2 (4)	C6—N1—C7—C12	64.1 (2)
C5—C1—C4—C3	177.3 (3)	C6—N1—C7—C8	−116.27 (17)
S1—C1—C4—C3	−3.7 (6)	C12—C7—C8—C9	−0.9 (2)
C2—C3—C4—C1	6.3 (6)	N1—C7—C8—C9	179.47 (14)
C4'—C1'—S1'—C2'	0.3 (4)	C12—C7—C8—C13	179.62 (15)
C5—C1'—S1'—C2'	−178.34 (14)	N1—C7—C8—C13	0.0 (2)
C1'—S1'—C2'—C3'	−6.5 (2)	C7—C8—C9—C10	−1.3 (3)
S1'—C2'—C3'—C4'	9.5 (4)	C13—C8—C9—C10	178.25 (16)
C5—C1'—C4'—C3'	−176.3 (3)	C8—C9—C10—C11	1.9 (3)
S1'—C1'—C4'—C3'	5.4 (7)	C9—C10—C11—C12	−0.4 (3)
C2'—C3'—C4'—C1'	−9.1 (7)	C10—C11—C12—C7	−1.6 (3)
C4'—C1'—C5—C6	112.7 (5)	C10—C11—C12—C14	176.81 (17)
S1'—C1'—C5—C6	−69.07 (18)	C8—C7—C12—C11	2.3 (2)
C4—C1—C5—C6	−78.1 (5)	N1—C7—C12—C11	−178.09 (15)
S1—C1—C5—C6	102.98 (15)	C8—C7—C12—C14	−176.12 (16)
C7—N1—C6—O1	−1.8 (2)	N1—C7—C12—C14	3.5 (2)

### Hydrogen-bond geometry (Å, °)

**Table:**

*D*—H···*A*	*D*—H	H···*A*	*D*···*A*	*D*—H···*A*
N1—H1···O1^i^	0.88	2.04	2.8701 (18)	157
C5—H5b···O1^i^	0.99	2.38	3.2622 (19)	148

Symmetry codes: (i) *x*−1, *y*, *z*.
